# Vendor-specific microbiomes influence oral cancer development and its response to *Streptococcus mitis* intervention in mice

**DOI:** 10.1080/20002297.2025.2611642

**Published:** 2026-01-05

**Authors:** Doaa E. El-Hadedy, Tsute Chen, Kathy Q. Cai, Andres J. Klein-Szanto, Anbin Mu, Kerry S. Campbell, Kelly A. Whelan, Nezar N. Al-Hebshi

**Affiliations:** a Department of Oral Health Sciences, Oral Microbiome Research Laboratory, Maurice H. Kornberg School of Dentistry, Temple University, Philadelphia, PA, USA; b The ADA Forsyth Institute, Somerville, MA, USA; c Histopathology Facility, Fox Chase Cancer Center, Philadelphia, PA, USA; d Fels Cancer Institute for Personalized Medicine, Lewis Katz School of Medicine, Temple University, Philadelphia, USA; e Cell Culture Facility, Fox Chase Cancer Center, Philadelphia, PA, USA; f Department of Cancer and Cellular Biology, Lewis Katz School of Medicine, Temple University, Philadelphia, USA; g Cancer Signaling and Microenvironment Program, Fox Chase Cancer Center, Temple University Health System, Philadelphia, USA; h Cancer Prevention and Control Program, Fox Chase Cancer Center, Temple University Health System, Philadelphia, USA

**Keywords:** Carcinogenesis, gastrointestinal microbiome, mice, mouth neoplasms, probiotic, *streptococcus mitis*

## Abstract

**Background:**

We previously demonstrated that *Streptococcus mitis* exhibits anticancer properties *in vitro*. Here, we sought to validate these findings *in vivo*. Because mice from different vendors harbor distinct microbiomes that can influence disease susceptibility and experimental outcomes, we also examined whether vendor-specific oral and gut microbiomes affect oral carcinogenesis and response to *S. mitis* intervention.

**Materials and methods:**

Oral carcinogenesis was induced using 4-NQO in C57BL/6 mice from Jackson Laboratory and Taconic Biosciences (*n* = 32 per vendor). Mice were randomized to biweekly oral swabbing with *S. mitis* or vehicle for 28 weeks. Oral and fecal microbiomes were profiled at baseline and week 8. At week 32, tongues were evaluated for tumor development.

**Results:**

Oral and gut microbiomes differed significantly between vendors. 4-NQO exposure induced marked microbial shifts and partial convergence of microbiome profiles. Jackson mice developed a significantly higher squamous cell carcinoma (SCC) burden. Several microbial taxa were associated with SCC, notably *Clostridium*, which was enriched in oral and fecal samples from Jackson mice. *S. mitis* reduced SCC burden in both cohorts and was accompanied by decreased *Clostridium* abundance.

**Conclusions:**

These data support *S. mitis* as a potential anticancer agent and underscore the importance of microbiome context in preclinical cancer models.

## Introduction

There is growing recognition of the role of microbiota in cancer development and progression, including in oral squamous cell carcinoma (OSCC)—a malignancy of the oral cavity associated with high morbidity and poor prognosis [1]. Dysbiosis of the oral or gut microbiota, or the presence of specific pathobionts, has been implicated in promoting carcinogenesis through mechanisms such as production of carcinogenic metabolites, sustaining proliferation and inducing chronic inflammation [2]. A prominent example is *Fusobacterium nucleatum*, which has been linked to both colorectal cancer and OSCC [3,4]. Conversely, the use of bacteria in cancer prevention or therapy has shown significant promise. Natural or engineered non-pathogenic bacterial species have been shown to selectively colonise tumours, induce tumour regression, serve as vectors for delivering therapeutic enzymes or proteins, and stimulate anti-tumour immune responses [5,6].

While the microbiota associated with OSCC has been extensively studied—including *in vitro* and animal models that mechanistically investigated the carcinogenic properties of pathobionts such as *F. nucleatum* and *Porphyromonas gingivalis*—the potentially protective or therapeutic role of health-associated oral bacteria in oral cancer remains relatively understudied. To address this, we recently screened six health-associated oral commensal for their effect on proliferation and global gene expression of OSCC cell lines [7]. Among them, *Streptococcus mitis*—a species consistently found to be depleted in healthy control samples in studies of periodontitis and OSCC [8–11]—demonstrated the most pronounced anticancer activity. *S. mitis* exhibited cytotoxicity against OSCC cell lines and induced transcriptional changes consistent with anticancer effects, including inhibition of the HOTAIR pathway, suppression of cell cycle progression through downregulation of cyclins, cyclin-dependent kinases, polo-like kinases, and metaphase signalling, as well as downregulation of pro-inflammatory pathways such as JAK/STAT, endothelin-1, HMGB1, and the acute phase response [7,12]. These promising *in vitro* findings warrant validation in an *in vivo* model of oral carcinogenesis.

To this end, the current study utilised the 4-nitroquinoline-1-oxide (4-NQO) mouse model of oral cancer—a well-established system that mimics the effects of exposure to tobacco in immunocompetent mice and recapitulates the molecular and histological spectrum of oral carcinogenesis in humans [13]. The model provides an excellent platform for longitudinal sampling and evaluating the role—and modulation—of the microbiome in oral tumorigenesis [14]. Mice of the same strain from different vendors (e.g. Jackson Laboratory and Taconic Biosciences) are known to harbour substantially different baseline oral and gut microbiomes [15–17]. These vendor-specific microbiota difference have been shown to influence experimental outcomes including disease severity in models of melanoma [18], malaria [19] and colitis [17] as well as response to treatment, for example response of melanoma to immunotherapy [18]. However, the impact of such baseline microbiota differences on 4-NQO-induced oral carcinogenesis has not been yet investigated.

The objective of this study was to evaluate the effect of *S. mitis* intervention on oral carcinogenesis in the 4-NQO model while assessing how vendor-specific microbiome differences influence cancer progression as well as response to *S. mitis*. We also characterised microbiome changes in the oral cavity and gut in response to 4-NQO exposure. To enable biologically relevant, high-resolution taxonomic classification, we used an optimised, mouse-specific 16S rRNA gene reference dataset.

## Methods

### Animals, housing conditions and experimental design

Sixty-four male and female C57BL/6 mice (6 weeks old) were purchased from two commercial vendors (*n* = 32 per vendor): Jackson Laboratory (Bar Harbour, ME) and Taconic Biosciences (Germantown, NY). Upon arrival, mice were housed under specific pathogen-free (SPF) conditions at Temple University’s animal facility, maintained at 22 ± 2 °C with a 12-hour light/dark cycle and provided with standard chow and drinking water *ad libitum*. Mice from the two vendors were housed in separate racks to minimise microbial cross-contamination.

**Figure 1.**
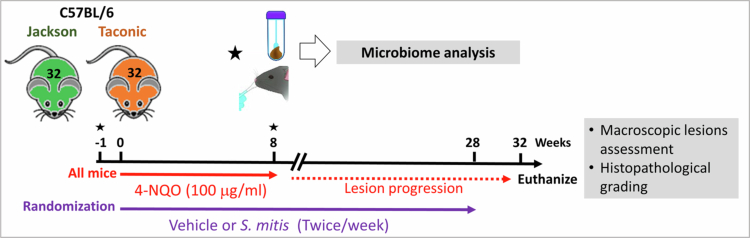
Study design. Six-week-old C57BL/6 mice from Taconic and Jackson laboratories (balanced by sex) were acclimated for one week, then randomised to receive vehicle or *Streptococcus mitis* (administered twice weekly). All mice were exposed to 100 µg/mL 4-nitroquinoline-1-oxide (4-NQO) in drinking water for 8 weeks to induce oral carcinogenesis, and followed up for an additional 24 weeks. Oral swab and faecal samples were collected at baseline (pre-4-NQO) and at week 8 (post-4-NQO) and were subjected to microbiome analysis by 16S rRNA gene sequencing. Bacterial intervention was discontinued at week 28. At the end of the follow-up period, mice were euthanized and tongues harvested for macroscopic and histopathological analysis.

The experimental design is depicted in Figure 1. After a one-week acclimatisation period, all mice received 4-NQO at a concentration of 100 μg/mL in their drinking water for 8 weeks to induce oral carcinogenesis, followed by a 24-week washout period to allow for lesion progression. Immediately upon initiation of 4-NQO treatment, mice were randomised equally by vendor and sex to receive either *S. mitis* intervention or vehicle control. The intervention continued for 28 weeks. Longitudinal oral and faecal samples were collected at baseline and week 8. At the end of the 32-week experiment, mice were euthanized, and tongues were harvested for macroscopic and histopathological evaluation.

The study was approved by Temple University Institutional Animal Care & Use Committee (Protocol # 5132).

### *S. mitis* preparation and administration

*S. mitis* ATCC 49456 was revived overnight from frozen stocks in Brain Heart Infusion (BHI) broth at 37 °C, sub-cultured the following morning and allowed to grow to mid-logarithmic phase (OD600 ~ 0.5). Fresh bacterial growth was then harvested, washed twice with sterile phosphate-buffered saline (PBS), and resuspended in 2% carboxymethylcellulose (CMC) in PBS to a final optical density OD_600_ of 0.125 (theoretically 10^8^ CFU/ml). For each mouse, a sterile polyester swab (Puritan, catalogue no. 25-806 2WD) was soaked in the bacterial suspension and used to apply approximately 100 μL of the inoculum to the tongue and buccal mucosa. Control mice were treated with swabs soaked in 2% CMC in PBS. Inoculations were performed twice per week, starting immediately after the initiation of 4-NQO treatment, for a total of 28 weeks.

### Sample collection

Oral and faecal samples were collected from the mice at baseline (prior to the initiation of 4-NQO treatment and *S. mitis* intervention) and at week 8. Oral samples were collected using sterile polyester mini-swabs (Puritan, catalogue no. 25-826 2WD) by applying several strokes to the dorsum of the tongue, rotating the swab 180°, and repeating the strokes to ensure adequate sampling. Swabs were then placed in sterile microcentifuge tubes. Faecal pellets were collected from individual mice by spontaneous defecation, stimulated by tapping on the abdomen, directly into sterile microcentrifuge tubes. When a mouse didn’t defecate, the blunt end of a dental paper point (size 80) was gently inserted into the rectum to collect a faecal sample. The tubes were not prefilled with preservative buffer; samples were immediately placed on ice and subsequently stored at −80 °C.

### Macroscopic and histopathological tongue evaluations

At the end of week 32, mice were euthanized and their tongues harvested. The tongues were immediately imaged using an M165C Stereo Microscope (Leica Microsystems, Wetzlar, Germany) before they were cut into two halves longitudinally; one half was formalin fixed and paraffin-embedded (FFPE) while the other half was flash frozen with liquid nitrogen. The images were later processed with ImageJ [20] to calculate lesion number, perimeter and total area. Longitudinal sections of tongue tissue (two sections per sample, 500 μm apart) were stained with Hematoxylin & Eosin (H&E) for histopathological evaluation of the lesions as hyperkeratosis, mild dysplasia, moderate dysplasia, moderate dysplasia, and invasive SCC. The percentage of the total epithelium occupied by each lesion type was documented.

### DNA extraction and sequencing

Genomic DNA from the oral swabs and faecal samples was extracted using the Invitrogen™ PureLink™ Microbiome DNA Purification Kit (Thermo Fisher Scientific, USA), following the manufacturer’s instructions. For faecal samples, 20 mg material from each sample was used. For the oral swabs, the lysis step was modified to maximise DNA yield. Specifically, smaller amounts of S1 and S2 buffers (600 μL and 75 μL instead of 800 μL and 100 μL, respectively) were added directly to the swab. The swab was then vigorously vortexed, transferred into a spin basket (Promega, USA) and centrifuged to recover the absorbed portion of lysate. The recovered lysate was pooled with the rest of the lysate (unabsorbed S1/S2 remaining in the tube) and used through the remainder of the protocol as per the manufacturer’s instructions. Six unused swabs were processed as negative extraction control. Extracted DNA was quantified using a NanoDrop spectrophotometer (Thermo Fisher Scientific, USA) and Qubit fluorometer (Thermo Fisher Scientific, USA), for the faecal and oral samples, respectively, and stored at −80 °C until further processing.

Prior to sequencing, DNA extracted from oral samples was pre-amplified with the degenerate primers 27FYM [21] and 519 R [22] targeting the V1-3 region of the 16S rRNA gene. Pre-amplification was performed with 25 PCR cycles in Platinum Superfi II PCR Master Mix (Thermo Fisher Scientific, USA). Nine negative controls were included: the six negative extraction controls mentioned above in addition to 3 PCR-grade water controls). Faecal DNA and pre-amplified oral DNA were then subjected to 16S rRNA gene sequencing using the same primer pair on the Illumina MiSeq platform with 2 × 300 bp paired-end chemistry. Sequencing was performed at the Integrated Microbiome Resource (IMR) in Halifax, Canada.

### Bioinformatic and statistical analysis

Resultant paired-end reads were merged with PEAR [23] and subsequently pre-processed—trimming, quality filtration, and chimera check—with mothur [24] as previously described [25]. High-quality, merged reads were then taxonomically assigned using a BLASTn-based algorithm [25,26] against a curated reference database specifically developed for this study. The database comprises 16S rRNA sequences from the mouse gut microbial biobank (mGMB) [27], the mouse oral microbiome database (https://momd.org/) [28], and NCBI 16S sequence database. The .fasta and .taxonomy files of this database are provided as Supplementary Dataset 1.

Potential contaminant taxa were identified based on their presence and relative abundance in negative control samples and were subsequently filtered out from the dataset. One major exception was *Cutibacterium acnes*, which appeared in both the negative controls and a subset of oral samples. However, qPCR analysis showed that its abundance was substantially higher in the oral samples than in the background (data not shown), so it was retained for downstream analyses.

The Phyloseq [29] and Microbiome packages in R [30] were used for alpha and beta diversity analysis. Significance of differences between groups in alpha diversity metrics was sought using t-test or Mann-Whitney test based on data distribution. For beta diversity, taxonomic read counts were centred log-ratio (CLR) transformed and used to compute Aitchison’s distances followed by principal component analysis (PCA). Pair-wise Adonis PERMANOVA (permutational analysis of variance) was used to assess significance of clustering, with *p*-values adjusted for multiple comparisons. MaAsLin3 (Microbiome Multivariable Associations with Linear Models 3) [31] package in R was used for differential abundance analysis by the experimental as well as outcome variables. CLR was used for data normalisation while false discovery rates (FDR) were set to 0.1 or 0.25 as appropriate.

## Results

### Jackson and taconic mice harbour distinct oral and gut microbiomes

**Figure 2.**
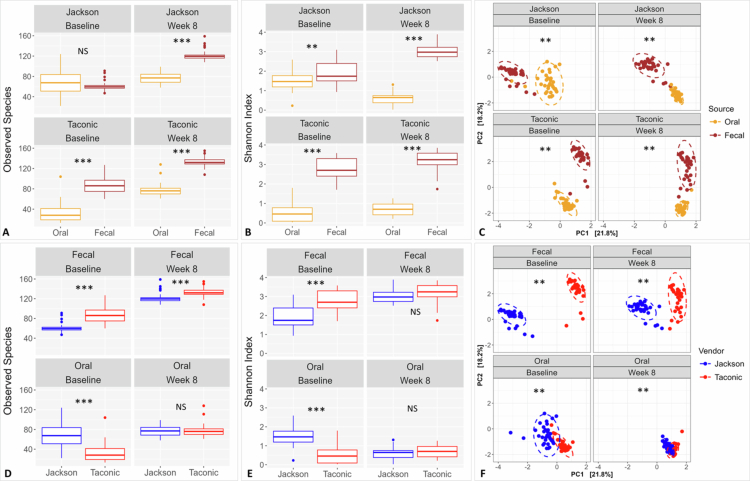
Microbiome diversity as a function of vendor and sample type. Rarified taxonomic profiles were used to calculate observed species (A, D) and Shannon’s alpha diversity index (B, E) for each variable (sample type or vendor), stratified by the other variable and by time point. Statistical significance was assessed with t-test or Mann-Whitney test, as appropriate. Principal Component Analysis (PCA) based on Aitchison distances computed from CLR-transformed data was performed to evaluate beta diversity across each variable, stratified by the other and by time point (C, F). Statistical significance of clustering was sought using pairwise PERMANOVA. The plots were generated with R. Adjusted p-values: ≤ 0.01 (**); ≤ 0.001 (***); NS, not significant.

Diversity analyses revealed marked differences both by sample type (oral vs. faecal) and vendor (Jackson vs. Taconic). Faecal samples showed significantly higher species richness and Shannon index (alpha diversity) than oral samples, and the two sample types clustered distinctly in PCA, irrespective of vendor and time point (Figure 2A−C). Beta diversity analysis further revealed significant separation by between Taconic and Jackson mice across sample types and time points (Figure 2F); however, differences in species richness and alpha diversity were most pronounced at baseline, being higher in Taconic mice for faecal samples and higher in Jackson mice for oral samples (Figure 2D-E).

**Figure 3.**
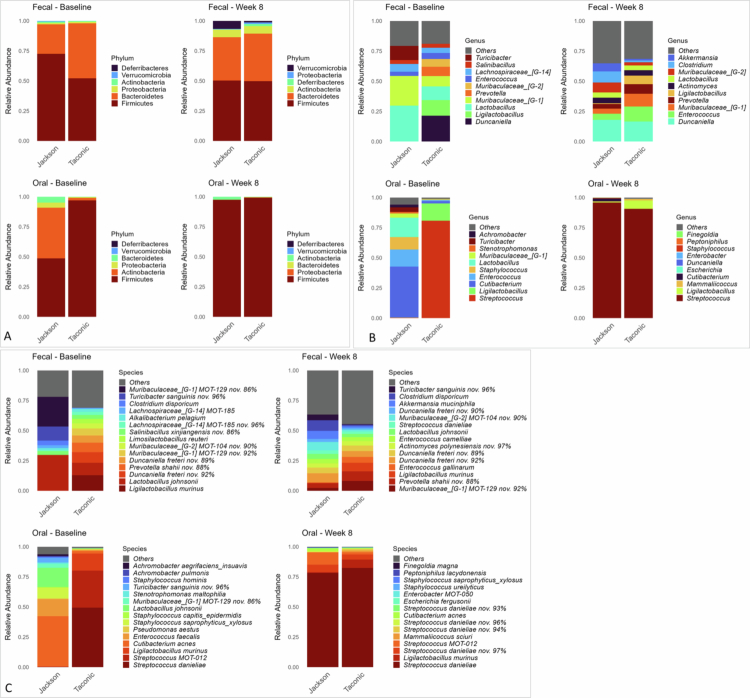
Microbial taxonomic profiles by vendor and sample type. DNA extracted from oral swab and faecal samples was subjected to 16S rRNA gene sequencing targeting the V1–V3 regions using paired-end chemistry. After merging and quality filtering, reads were taxonomically classified using BLASTn against a curated reference database of mouse oral and gut 16S rRNA gene sequences. Stacked bar plots display average relative abundances across samples at (A) the phylum level (all identified phyla), (B) the genus level (top 10 genera), and (C) the species level (top 15 species), stratified by vendor, sample type and time point (4-NQO treatment). Plots were generated in R.

The corresponding microbial profiles illustrating the differences between oral and faecal samples, as well as between Jackson and Taconic mice, at three taxonomic levels are presented in Figure 3. Faecal samples were dominated by the phyla Firmicutes and Bacteroidetes with an expansion of Actinobacteria and Verrucomicrobia by week 8. At the genus level, the most abundant taxa overall were *Duncaniella, Ligilactobacillus, Lactobacillus, Muribaculaceae (G-1 and G-2), Enterococcus, Prevotella and, Lachnospiraceae G-14* with an increased representation of *Clostridium* and *Akkermansia* at week 8. However, the average relative abundances of these genera varied by vendor and over time. Notably, Jackson mice had a distinct baseline microbiome with negligible levels of *Duncaniella* but markedly higher abundance of *Lactobacillus* and *Turicibacter.* Similar differences in microbial profiles were evident at the species level. Notably, approximately 44% of faecal sample sequences matched known species in the reference data set predominantly *Lactobacillus johnsonii*, *Ligilactobacillus murinus*, *Clostridium disporicum*, *Enterococcus camelliae*, *Akkermansia muciniphila.* The remaining reads were clustered *de novo* using USEARCH [32] into operational taxonomic units (OTUs) which were treated as potentially novel species. Each OTU was annotated based on its closest known match using percent identity, such that a designation like ‘*Duncaniella freteri* nov. 92%’ indicates a putative novel species most closely related to *Duncaniella freteri*, sharing 92% sequence identity.

Oral samples were overwhelmingly dominated by the phylum *Firmicutes*, primarily represented by genus *Streptococcus* with appreciable contributions from *Ligilactobacillus*. This pattern was consistent across subgroups with one striking exception: Jackson mice at baseline exhibited a distinct oral microbiome characterised by high relative abundances of Actinobacteria—particularly *Cutibacterium*, *Staphylococcus*, and *Enterococcus*—as well as *Lactobacillus*, while *Streptococcus* was virtually undetectable. Approximately, 93% of oral sample reads were assigned to known species. Genus *Streptococcus* was primarily represented by *Streptococcus danieliae* and, to a lesser extent, *Streptococcus* mouse oral taxon (MOT) 012, which along with *L. murinus,* constituted the majority of the oral microbiota except in Jackson baseline samples. The latter were dominated with *Cutibacterium acnes*, *L. johnsonii, Enterococcus faecalis* and *Staphylococcus saprophyticus/xylosus.*

There were also significant differences in microbial diversity and profiles by sex, which are presented in Supplementary Figures 1−3.

### 4-NQO treatment induces convergence of microbiomes between Jackson and Taconic mice

**Figure 4.**
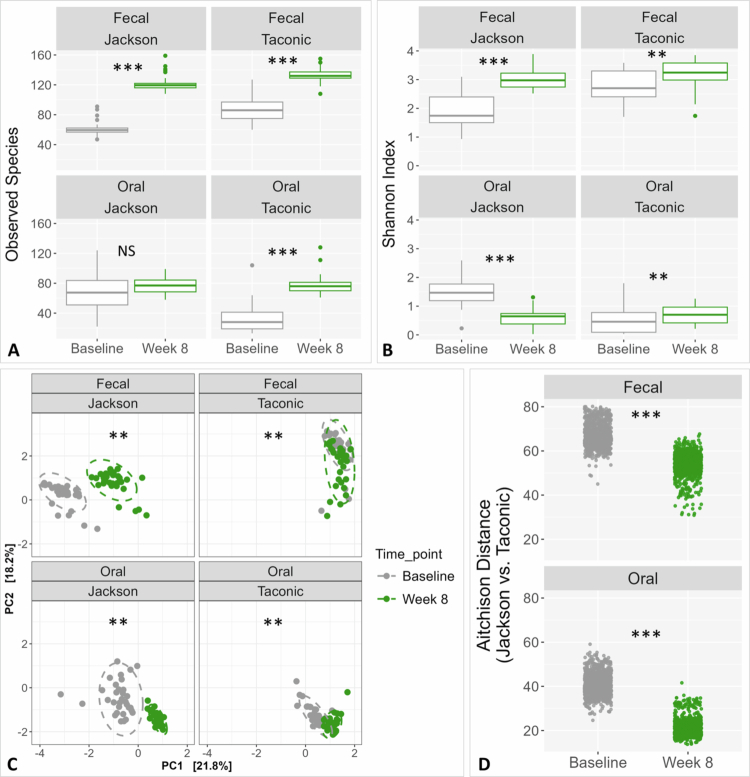
Microbiome diversity changes and convergence induced by 4-NQO treatment. Rarified taxonomic profiles were used to calculate observed species (A) and Shannon’s alpha diversity index (B) for each time point stratified by vendor and sample type. Statistical significance was assessed with t-test or Mann-Whitney test, as appropriate. Principal Component Analysis (PCA) based on Aitchison distances computed from CLR-transformed data was performed to evaluate beta diversity across time points (4-NQO treatment) stratified by vendor and sample type. Statistical significance of clustering was sought using PERMANOVA. (D) Inter-group Aitchison distances between Jackson and Taconic mice were calculated and plotted to assess convergence in oral and faecal microbiomes following 4NQO treatment. A significant reduction in distances after treatment indicates increased similarity in microbial composition between the two vendor groups. Statistical significance was assessed with t-test. The plots were generated with R. Adjusted *p*-values: ≤ 0.01 (**); ≤ 0.001 (***); NS, not significant.

4-NQO treatment (baseline vs. week 8 samples) resulted in significant microbial diversity changes. In faecal samples, 4-NQO increased both species richness and Shannon diversity index, whereas in oral samples, it increased species richness but reduced Shannon index (Figure 4A-B). Beta diversity analysis revealed significantly separation between baseline and week 8 samples across sample type and vendor (Figure 4C). However, inter-group Aitchison distances between Jackson and Taconic mice significantly decreased from baseline to week 8 in both oral and faecal samples, indicating a convergence of the microbiomes in response to 4-NQO treatment (Figure 4D).

### *S. mitis* is cleared by mice but results in modest, vendor-specific microbiome diversity changes

While preliminary experiments demonstrated that *S. mitis* successfully colonised the oral cavity one week after inoculation (data not shown), it was hardly identified in the sequencing data in samples collected at week 8 as confirmed by qPCR analysis (data not shown), indicating that *S. mitis* was cleared by the mice.

**Figure 5.**
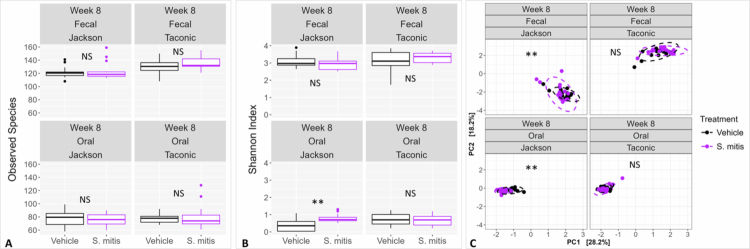
Microbiome diversity changes in response to *S. mitis* treatment. Rarified taxonomic profiles at week 8 were used to calculate observed species (A) and Shannon’s alpha diversity index (B) within the control and *S. mitis*-treated groups, stratified by vendor and sample type. Statistical significance was assessed with t-test or Mann-Whitney test, as appropriate. Principal Component Analysis (PCA) based on Aitchison distances computed from CLR-transformed data was performed to evaluate beta diversity across treatment groups, also stratified by vendor and sample type (C). Statistical significance of clustering was sought using PERMANOVA. The plots were generated with R. Adjusted *p*-values: ≤ 0.01 (**); NS, not significant. The plots were generated with R.

Nevertheless, comparison of intervention and vehicle control samples at week 8 revealed that *S. mitis* induced microbiome changes, particularly in Jackson mice. Specifically, oral samples from *S. mitis*–treated Jackson mice exhibited a modest but significant increase in Shannon diversity index. In addition, beta diversity analysis showed significant differences between intervention and control groups for both oral and faecal samples in Jackson mice, though the degree of separation remained minimal (Figure 5).

### Microbiome composition is primarily shaped by sample type, vendor origin and 4-NQO exposure

**Figure 6.**
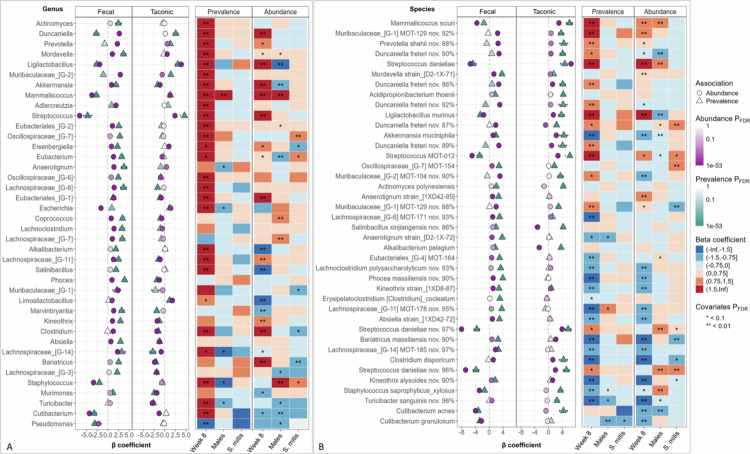
Modelling of differentially abundant and prevalent microbial taxa by experimental variables. Centred log ratio (CLR) transformed data were analysed with MaAsLin3 package in R to identify differentially abundant or prevalent genera across experimental variables. Fixed effects included vendor (Taconic vs. Jackson), sample type (faecal vs. oral), 4NQO treatment (week 8 vs. baseline), sex (male vs. female), and bacterial intervention (*S. mitis* vs. vehicle). Features with a false discovery rate (FDR) ≤ 0.1 were considered significant. The top 40 genera (A) and top 40 species (B) are shown. For the two variables with the strongest associations, results are visualised as coefficient plots; associations with the remaining variables are displayed as heat maps. MOT: Mouse Oral Taxon. Nov.: Potentially novel species, followed by percent identity to the closest species in the reference data set.

MaAsLin3 was used to identify microbial features independently associated with each of the experimental variables. Among the variables included in the model, sample type had the strongest association with microbiome composition, followed by vendor origin and 4-NQO exposure. The top differentially abundant and/or prevalent genera are presented in Figure 6A. Oral samples were enriched in *Streptococcus*, *Cutibacterium*, *Staphylococcus*, and *Mammaliicoccus*, whereas faecal samples were substantially enriched in *Lachnospiraceae* G-14, *Muribaculaceae* G-1 and G-2, *Duncaniella*, *Adlercreutzia*, and *Prevotella*. Vendor-associated difference included higher levels of *Ligilactobacillus*, *Streptococcus*, *Limosilactobacillus*, *Magnetovibrio*, and *Lachnospiraceae* G-14 in Taconic mice and enrichment of *Kineothrix*, *Turicibacter*, *Bariatricus*, *Murimonas*, *Clostridium*, and *Lachnospiraceae* G-3 in Jackson mice.

4-NQO treatment was associated with increased abundance and/or prevalence of *Streptococcus*, *Akkermansia*, *Mammaliicoccus*, *Faecalibaculum*, *Duncaniella*, and *Clostridium*, and decreased abundance and/or prevalence of *Pseudomonas*, *Marvinbryantia*, and *Murimonas*. Fewer associations were detected by sex, with *Gluceribacter*, *Lachnospiraceae* G-7, *Staphylococcus*, and *Mammaliicoccus* enriched in males, and *Lactobacillus*, *Ligilactobacillus*, *Akkermansia*, and *Cutibacterium* enriched in females. *S. mitis* intervention showed the weakest association with microbial composition—the results are elaborated upon in a separate section below.

The corresponding results at the species level, without further elaboration, are shown in Figure 6B. Exhaustive lists of differentially abundant and prevalent genera and species are provided in Supplementary files 1−2. To further dissect variable-specific effects, MaAsLin3 analyses were also conducted independently for each variable, stratified by the remaining variables; results are presented in Supplementary Figures 4−13.

### Higher SCC burden in Jackson mice is associated with vendor-specific microbiome features, notably *Clostridium*

At the end of the 32-week experiment, mice were euthanized and their tongues were harvest for macroscopic evaluation (assessed in terms of lesion count and size metrics) and histopathological examination (reported as proportion of epithelium occupied by dysplasia or SCC). Despite comparable macroscopic lesion parameters between the vendor groups, Jackson mice exhibited a significantly higher burden of SCC compared to Taconic mice (Table 1). Specifically, the mean percentage of SCC in Jackson mice was 6.23% versus 0.96% in Taconic mice (*P* < 0.001). No significantly differences in the other histopathological features were observed.

**Table 1.** Macroscopic and histopathological of tongues harvested at the end of the experiment (32 weeks).

Variable	Total (*n* = 45)	Jackson (*n* = 22)	Taconic (*n* = 23)	Females (*n* = 21)	Males (*n* = 24)
No. of lesions	1.80 ± 1.20	1.64 ± 0.95	1.96 ± 1.40	1.76 ± 1.00	1.83 ± 1.37
Lesion Area (mm^2^)	4.38 ± 4.29	4.19 ± 4.16	4.56 ± 4.49	2.95 ± 3.31	5.64 ± 4.70
Lesion Perimeter (mm)	9.81 ± 6.76	8.80 ± 5.28	10.77 ± 7.93	7.99 ± 5.28	11.40 ± 7.59
% Mild Dysplasia	18.42 ± 6.96	18.41 ± 7.06	18.43 ± 7.03	18.00 ± 6.25	18.79 ± 7.64
% Moderate Dysplasia	10.11 ± 8.30	11.41 ± 9.40	8.87 ± 7.09	12.71 ± 10.11	7.83 ± 5.62
% Severe Dysplasia	1.13 ± 1.89	1.23 ± 2.02	1.04 ± 1.80	0.90 ± 2.05	1.33 ± 1.76
% Total dysplasia	29.67 ± 11.78	31.05 ± 11.11	28.35 ± 12.49	31.62 ± 11.13	27.96 ± 12.30
% SCC	3.53 ± 6.21	6.23 ± 7.62*	0.96 ± 2.74*	4.10 ± 5.61	3.04 ± 6.77

* *P* < 0.001, t-test. SCC: Squamous cell carcinoma.

**Figure 7.**
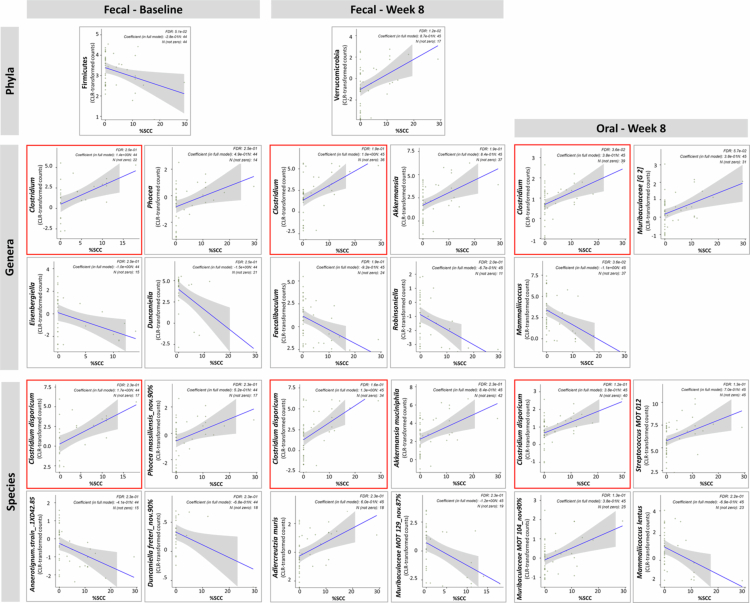
Microbiome features associated with squamous cell carcinoma (SCC) burden. Centred log-ratio (CLR) transformed taxonomic data were analysed using the MaAsLin3 package in R to identify microbial phyla, genera, and species significantly associated with SCC burden, quantified as the percentage of epithelium exhibiting SCC based on histopathological evaluation. Correlation plots are shown for representative taxa at each taxonomic level, presented separately for each sample type and time point combination—no significant associations were observed with the oral microbiome at baseline. Vendor was excluded from the models to avoid collinearity. Taxa with a false discovery rate (FDR) ≤ 0.25 were considered significant.

MaAsLin3 analysis, stratified by sample type and time point, identified several microbial features in association with SCC burden, as shown in Figure 7. Among these, increased abundance of the genus *Clostridium*—particularly *Clostridium disporicum*— was consistently associated with SCC burden across most stratified analyses (baseline and week 8 faecal samples, and week 8 oral samples). Other notable associations included increased *Phocea* and decreased *Duncaniella* in baseline faecal samples; increased *Akkermansia* and decreased *Faecalibaculum* in week 8 faecal samples; and increased *Muribaculaceae G-2* and decreased *Mammaliicoccus* in week 8 oral samples. No associations were found in baseline oral samples.

**Figure 8.**
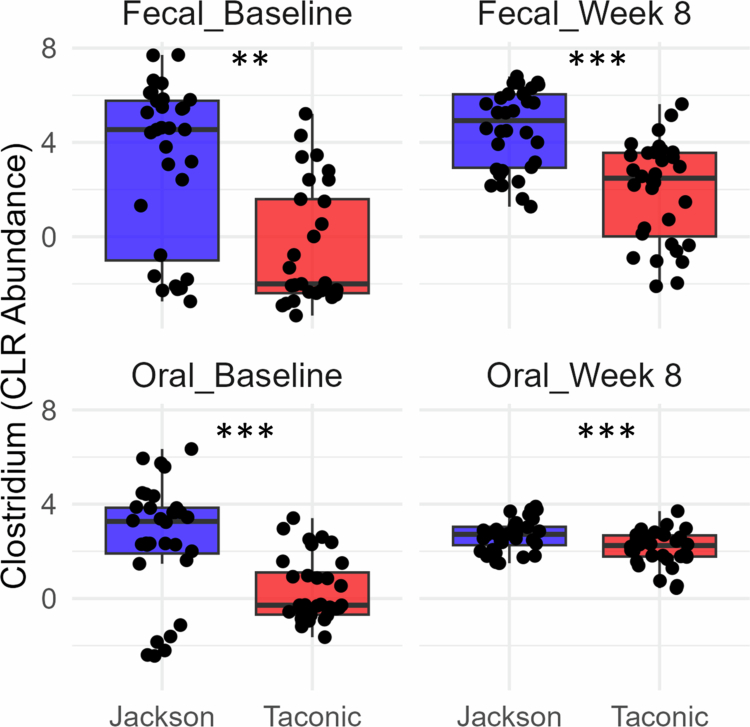
Abundances of *Clostridium* in the microbiomes of Jackson and Taconic mice. Boxplots display CLR-transformed abundances of genus *Clostridium* across Jackson and Taconic mice, stratified by sample type and time point. Statistical significance of differences was determined based on false discovery rate (FDR) from MaAsLin3. FDR: ≤ 0.01 (**); ≤ 0.001 (***).

Notably, vendor origin was excluded from the MaAsLin3 models above evaluating SCC-associated microbiota to avoid collinearity. In subsequent follow-up analysis of abundance data, *Clostridium* was found to be significantly more abundant in Jackson mice across sample types and time points (Figure 8), suggesting that vendor-specific enrichment of this taxon may underlie the greater SCC burden observed in Jackson mice.

**Figure 9.**
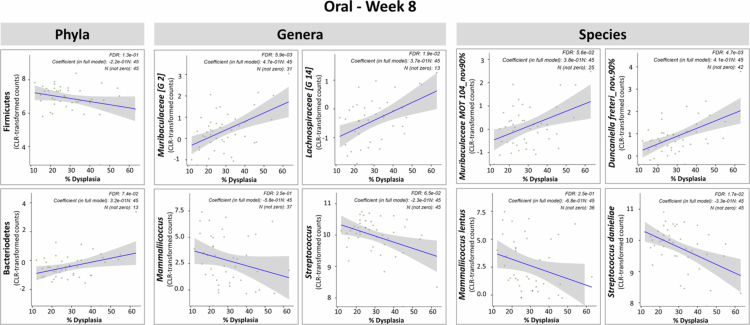
Microbiome features associated with dysplasia burden. Centred log-ratio (CLR) transformed taxonomic data were analysed using the MaAsLin3 package in R to identify microbial phyla, genera, and species significantly associated with dysplasia burden, quantified as the percentage of epithelium exhibiting mild, moderate and/or severe dysplasia based on histopathological evaluation. Correlation plots are shown for representative taxa at each taxonomic level. Significant associations were only observed with the oral microbiome at week 8. Vendor was excluded from the model to avoid collinearity. Taxa with a false discovery rate (FDR) ≤ 0.25 were considered significant.

We performed a similar analysis for total dysplasia burden, which revealed significant associations only in oral samples at week 8 (Figure 9). Notably, *Muribaculaceae G-2* was positively associated with dysplasia burden, while *Mammaliicoccus*—particularly *Mammaliicoccus lentus*—showed an inverse association. These patterns are consistent with their respective associations with SCC observed in the same sample type and time point.

### *S mitis* reduces SCC burden in both Taconic and Jackson mice, possibly through microbiome modulation

To assess the influence of *S. mitis* intervention on tumour outcomes, we compared the macroscopic and histopathological findings between *S. mitis*-treated and vehicle control mice within each vendor group (Table 2). Although no statistically significant differences were observed in terms of lesion count and size or dysplasia burden, *S. mitis* administration resulted in a statistically significant reduction in SCC burden in both Jackson (5.63% vs. 7.83%, *P* = 0.037) and Taconic (0.43% vs. 1.78%, *P* = 0.023) mice.

**Table 2.** The effect of *S. mitis* intervention on macroscopic and histopathological outcomes.

Variable	Jackson			Taconic		
	Vehicle	*S. mitis*	*P*	Vehicle	*S. mitis*	*P*
No. of lesions	1.17 ± 0.75	1.81 ± 0.98	0.289	2.67 ± 1.50	1.50 ± 1.16	0.254
Lesion Area (mm^2^)	3.93 ± 4.22	4.30 ± 4.28	0.76	4.57 ± 3.87	4.56 ± 4.99	0.295
Lesion Perimeter (mm)	7.05 ± 5.03	9.45 ± 5.38	0.678	13.02 ± 7.54	9.32 ± 8.12	0.527
% Mild Dysplasia	13.17 ± 4.62	20.38 ± 6.89	0.622	16.44 ± 3.21	19.71 ± 8.53	0.205
% Moderate Dysplasia	10.33 ± 12.16	11.81 ± 8.60	0.506	4.89 ± 6.19	11.43 ± 6.58	0.660
% Severe Dysplasia	2.00 ± 1.10	0.94 ± 2.24	0.226	0.44 ± 0.88	1.43 ± 2.14	0.094
% Total dysplasia	25.50 ± 10.25	33.13 ± 10.99	0.6	21.78 ± 7.38	32.57 ± 13.47	0.105
% SCC	7.83 ± 11.65	5.63 ± 5.88	0.037*	1.78 ± 4.06	0.43 ± 1.34	0.023*

* t-test. SCC: Squamous cell carcinoma.

**Figure 10.**
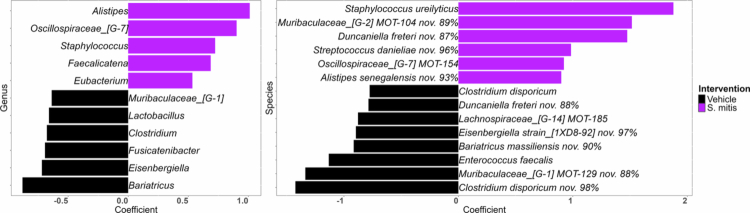
Differentially abundant microbial taxa in response to *S. mitis* intervention. CLR-transformed taxonomic data were analysed using MaAsLin3, controlling for other experimental variables, to identify microbial genera and species associated with *S. mitis* treatment. The contrast focused on *S. mitis* vs. vehicle at week 8. Coefficient barplots display representative taxa with a false discovery rate (FDR) ≤ 0.1. The plots were generated with R.

Microbiome composition shifts associated with *S. mitis* intervention, which were identified through the MaAsLin3 modelling described earlier, are presented in Figure 10. Several differentially abundant genera and species were observed between *S. mitis*-treated and control mice. Notably, *S. mitis* intervention was associated with a reduction in the abundance of *Clostridium*, a genus shown above to be associated with SCC, which suggests that *S. mitis* mediates it protective effect in part by microbiome modulation.

## Discussion

This study provides the first *in vivo* evidence that *S. mitis,* an oral bacterial commensal previously demonstrated to have anticancer properties *in vitro* [7,12], can reduce tumour burden in a murine model of oral cancer. To our best knowledge, this is also the first to simultaneously profile both oral and gut microbiomes in the context of 4-NQO–induced oral carcinogenesis, and to demonstrate how vendor-specific differences (i.e. Jackson vs. Taconic) in these microbiomes correlate with tumour susceptibility. By incorporating longitudinal sampling, the study also revealed how 4-NQO exposure affects microbiome composition across anatomical sites. An additional novel aspect of this study is the development of a curated, 16S rRNA reference data set, in which a substantial proportion of sequences are derived from bacteria isolated from mice. This allowed biologically-relevant, species-level taxonomy resolution.

The oral and faecal microbiomes were completely distinct, with little overlap in predominant taxa, emphasising that the oral cavity and the gut are separate ecological niches with distinct environmental factors. This is true despite the common practice of coprophagy in mice, which would otherwise be expected to introduce some overlap between the two sites. Studies that directly compared the oral and gut microbiomes in mice are nearly lacking— one such example exists in BALB/c mice [33]—and, to our knowledge, no such data are available for C57BL/6 mice. Nevertheless, the marked differences in microbial composition between these anatomical sites are evident when comparing results across studies that focused individually on either the oral or gut microbiomes [15,34,35].

We also found striking differences in microbiome composition based on vendor origin, consistent with previous reports for both oral and faecal sites. For example, Abusleme et al. [15]. identified distinct microbial profiles between Jackson and Taconic C57BL/6 mice at 10, 32 and 52 weeks of age. Notably, Jackson mice had substantially higher microbial diversity, consistent with our results at baseline. Ericsson et al. [17]. reported marked differences in faecal microbiomes between Harlon and Jackson mice, across three mouse strains and four time points, with Harlon mice having greater richness and alpha diversity. However, some studies reported less subtle differences. Similarly, Caruso et al. [16]. found significant differences in faecal microbiomes between Jackson and Taconic mice from the age of 3 weeks through 28 days post-weaning. Importantly, their study demonstrated that cohousing mice from the two vendors led to convergence of microbiomes.

Comparing microbial profiles across studies is challenging due to methodological variations. These include differences in the age of mice studied, sample type and collection method, DNA extraction protocol, sequencing approach and bioinformatic analysis. Notably, the latter steps— particularly, the choice of 16S rRNA gene hypervariable region, sequencing primers, taxonomy assignment algorithm and reference database—can substantially affect taxonomical resolution. This is particularly true for gut microbiome studies, where many reports present only family-level data or genus-level profiles with a large fraction of unclassified reads [16,17,34,35]. Oral microbiome studies tend to report higher taxonomic resolution, including species-level identification [15], which has recently been facilitated by development of the Mouse Oral Microbiome Database (MOBD)—a key component of the reference database used in the current study. In addition to these methodological variation, cross-study comparisons are further complicated by ongoing to bacterial taxonomy and nomenclature.

Nevertheless, some consistencies can be observed. For example, several of the major genera identified in faecal samples in the current study—such as *Lactobacillus* (including *Ligilactobacillus*), *Duncaniella*, *Muribaculaceae*, *Turicibacter*, *Prevotella*, and members of the *Lachnospiraceae* family—have also been reported as abundant taxa in one or more previous studies [33–36]. The dominance of streptococci in murine oral samples, particularly S. *danieliae,* have also been previously reported [15]. Although an oral microbiome dominated by *Cutibacterium*, *Enterococcus*, and *Staphylococcus*, as observed in Jackson mice at baseline in this study, is not typical of most published reports, it is not without precedent. For example, Wu et al. reported elevated levels of *Enterococcus* and *Staphylococcus* in the oral microbiota of both germ-free mice following colonisation and specific-pathogen free mice [37]. In our study, these taxa were nearly absent in Taconic mice at both time points and declined markedly in Jackson mice by week 8. Together with the qPCR quantification of *Cutibacterium acnes* and comparisons with negative controls (see Methods), these findings strongly rule out environmental contamination. Nevertheless, it is likely that these taxa represent transient rather than resident member of the murine oral microbiome.

The differences in the oral and gut microbiomes between Taconic and Jackson mice may explain, at least in part, the higher SCC burden observed in Jackson mice. Several taxa were significantly associated with SCC burden, notably the genus *Clostridium*. In particular, *Clostridium* showed consistent positive associations with SCC burden in faecal samples at both baseline and week 8, as well as in oral samples at week 8. Moreover, *Clostridium* was consistently more abundant in Jackson mice across all sample types and time points. Notably, *C. disporicum* has recently been identified in OSCC human samples [38] and implicated in colorectal cancer [39]. Whether or not *C. disporicum* plays a role in cancer progression needs to be explored.

The *S. mitis* intervention involved biweekly inoculations over 28 weeks. Surprisingly, *S. mitis* was barely detectable in week 8 samples, indicating that it failed to establish persistent colonisation. Possible explanations include competition by intrinsic mouse microbiota or clearance by host immune defenses. Despite this transient presence, *S. mitis* intervention resulted in lower SCC burden in both Jackson and Taconic mice. How *S. mitis* exerts such anticancer actions *in vivo* remain unclear and was not been addressed mechanistically in this study. One possibility is direct cytotoxicity and anti-proliferative effects as demonstrated in our previous *in vitro* studies [7,12]. Additionally, *S. mitis* may have stimulated an antitumor immune response. A third possibility is that *S. mitis* induced protective microbiome changes. For example, *S. mitis* administration was associated with reduced abundance of the genus *Clostridium*, including *C. disporicum*, which was consistently associated with higher cancer burden across sample types and time points. Interestingly, members of the mitis streptococci have been shown to inhibit *F. nucleatum* and interfere with its inflammatory properties through H_2_O_2_ production [40,41], suggesting this could be additional mechanisms *S. mitis* play anticancer properties. Overall, these findings corroborate the expanding literature on the potential use of streptococci or their components in cancer therapy [42], but further work to identify mechanisms underlying *S. mitis* anticancer properties are required.

The 4-NQO model is widely used to study oral carcinogenesis, as it replicates the stepwise progression and molecular features of OSCC in humans [13]. This has been confirmed by recent genomic, transcriptomic and immunological studies [43,44]. The model is also well-suited for investigating the role of specific microbes or microbiome perturbations in the development of oral cancer [45]. However, as with all models, 4-NQO has its limitations, including slow tumour induction and progression, protocol dependence, and high heterogeneity in terms of lesion number, distribution and histopathological severity [46,47]. The latter represents a major challenge to estimation of SCC burden, which is typically assessed based on histopathological analysis of representative sections rather than exhaustive evaluation of all lesions. Accordingly, the model does not provide controlled tumour growth suitable for standardised testing of therapeutic interventions. Given our focus on microbiome–carcinogen interactions and longitudinal microbial profiling during initiation and progression, the 4-NQO model aligns well with the study’s biological question; however, follow-up validation studies in complementary systems, such as syngeneic transplant models [48], are warranted.

In conclusion, this study highlights the differences in both oral and gut microbiome composition between mice from different vendors and how that can affect oral carcinogenesis. We identified specific microbial taxa, including *Clostridium disporicum*, that were enriched in Jackson mice and consistently associated with increased tumour burden. Conversely, intervention with *S. mitis*, despite its transient colonisation, significantly reduced SCC burden. These findings illustrate the complex interplay between the microbiome and cancer development, while supporting the growing interest in the use of commensal bacteria as therapeutic or preventive strategies. Future studies are needed to assess the potential carcinogenic properties of specific microbes such as *C. disporicum*, dissect the *in vivo* anticancer mechanisms of *S. mitis* and explore its translational potential in oral cancer prevention and therapy.

## Supplementary Material

Supplementary figures.pdf Supplementary Figures.pdf

## Data Availability

The 16S rRNA gene sequencing data generated in this study have been deposited in Sequence Read Archive (https://www.ncbi.nlm.nih.gov/sra/) under the accession number PRJNA1289179.
