# Supplementary material for: Vendor-specific microbiomes influence oral cancer development and its response to Streptococcus mitis intervention in mice
Source: J Oral Microbiol. 2026 Jan 5;18(1):2611642. doi: 10.1080/20002297.2025.2611642 (PMC12777837; doi:10.1080/20002297.2025.2611642)
Supplement: Supplementary figures.pdf [file ZJOM_A_2611642_SM6180.pdf]

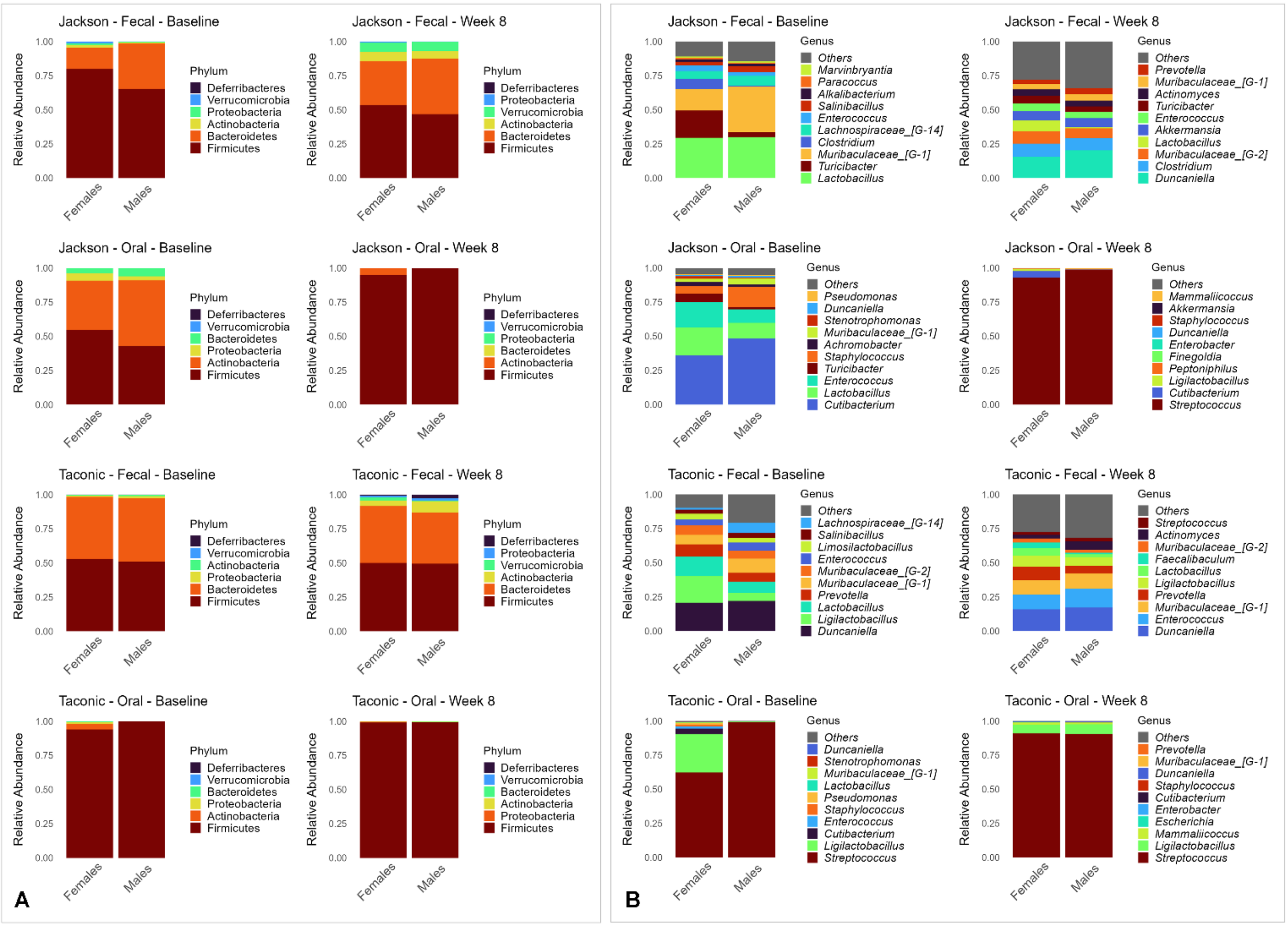

Supplementary Figure 1. Phylum-level (A) and genus-level (B) taxonomy profiles by sex stratified by vendor, sample type, and 4-NQO treatment (time points). DNA extracted from oral swab or fecal samples was sequenced for V1-V3 regions of the 16S rRNA gene using paired-end chemistry. Merged, quality-filtered were assigned taxonomies using a BLASTn-based algorithm. Stacked bars represent the average relative abundances of all the identified phyla and top 10 genera across samples. The plots were generated with R.

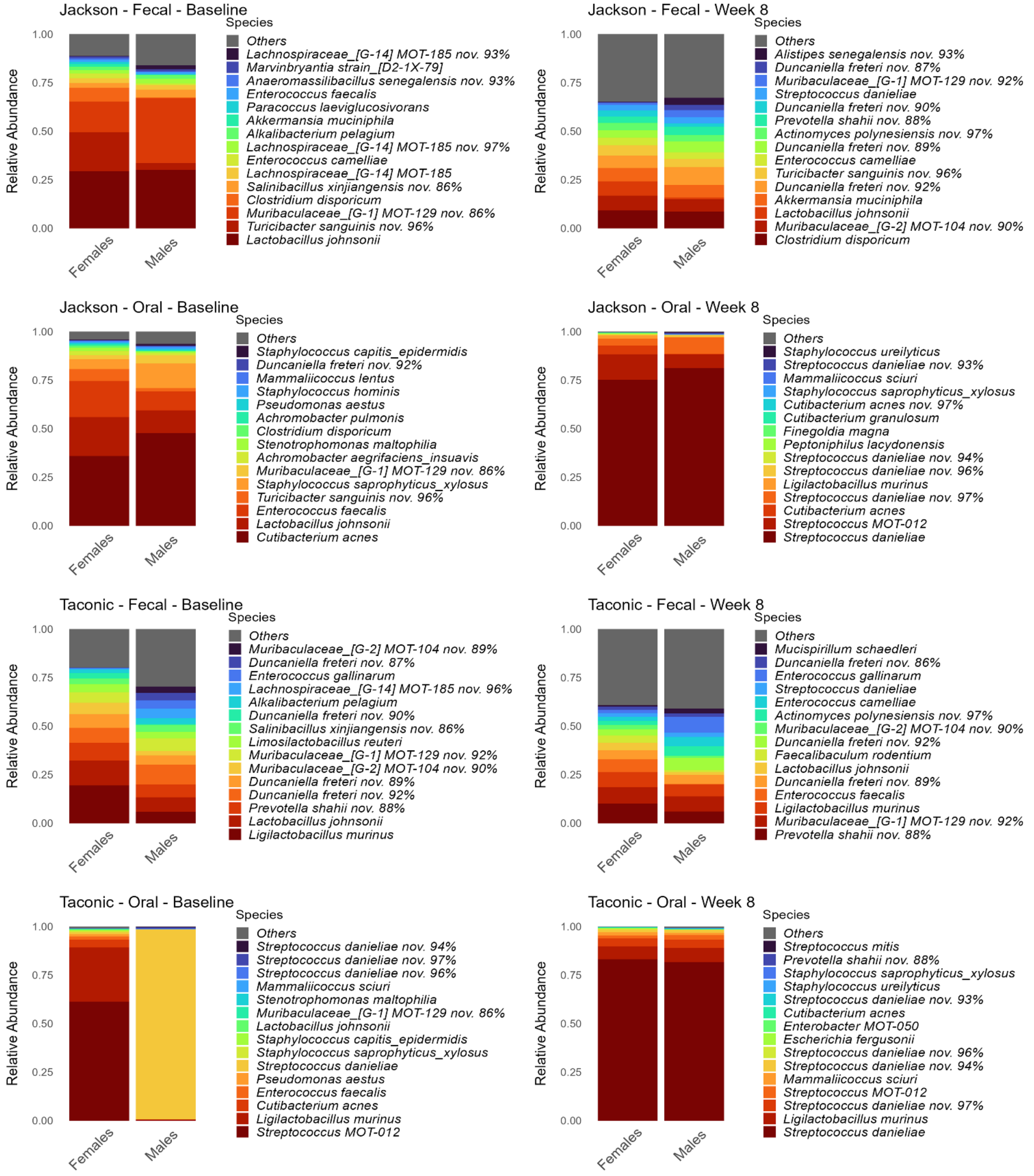

Supplementary Figure 2. Species-level taxonomy profiles by sex stratified by vendor, sample type, and 4-NQO treatment (time points). DNA extracted from oral swab or fecal samples was sequenced for V1-V3 regions of the 16S rRNA gene using paired-end chemistry. Merged, quality-filtered were assigned taxonomies using a BLASTn-based algorithm. Stacked bars represent the average relative abundances of top 15 species across samples. The plots were generated with R.

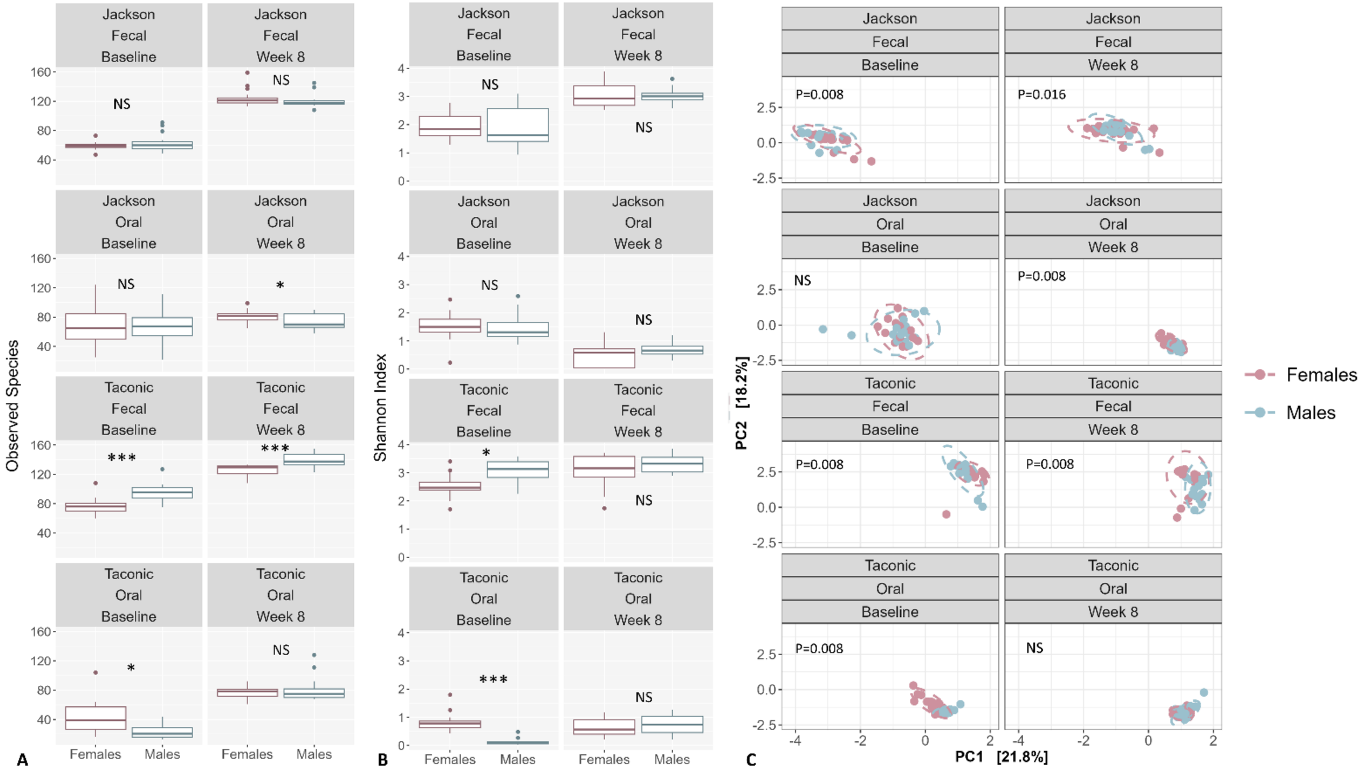

Supplementary Figure 3. Species richness, alpha and beta-diversity by sex stratified by vendor, sample type, and 4-NQO treatment (time points). Rarified taxonomic profiles were used to calculate observed species (A) and Shannon's alpha diversity index (B). Statistical significance was assessed with t-test or Mann-Whitney test as appropriate. * Adjusted p-value ≤ 0.05; *** Adjusted p-value ≤ 0.001; NS, not significant. Principle Component Analysis (C) was performed using Aitchison distances calculated from centered log-ratio (CLR) transformed taxonomic profiles. Statistical significance was sought using PERMANOVA; NS, not significant. The plots were generated with R.

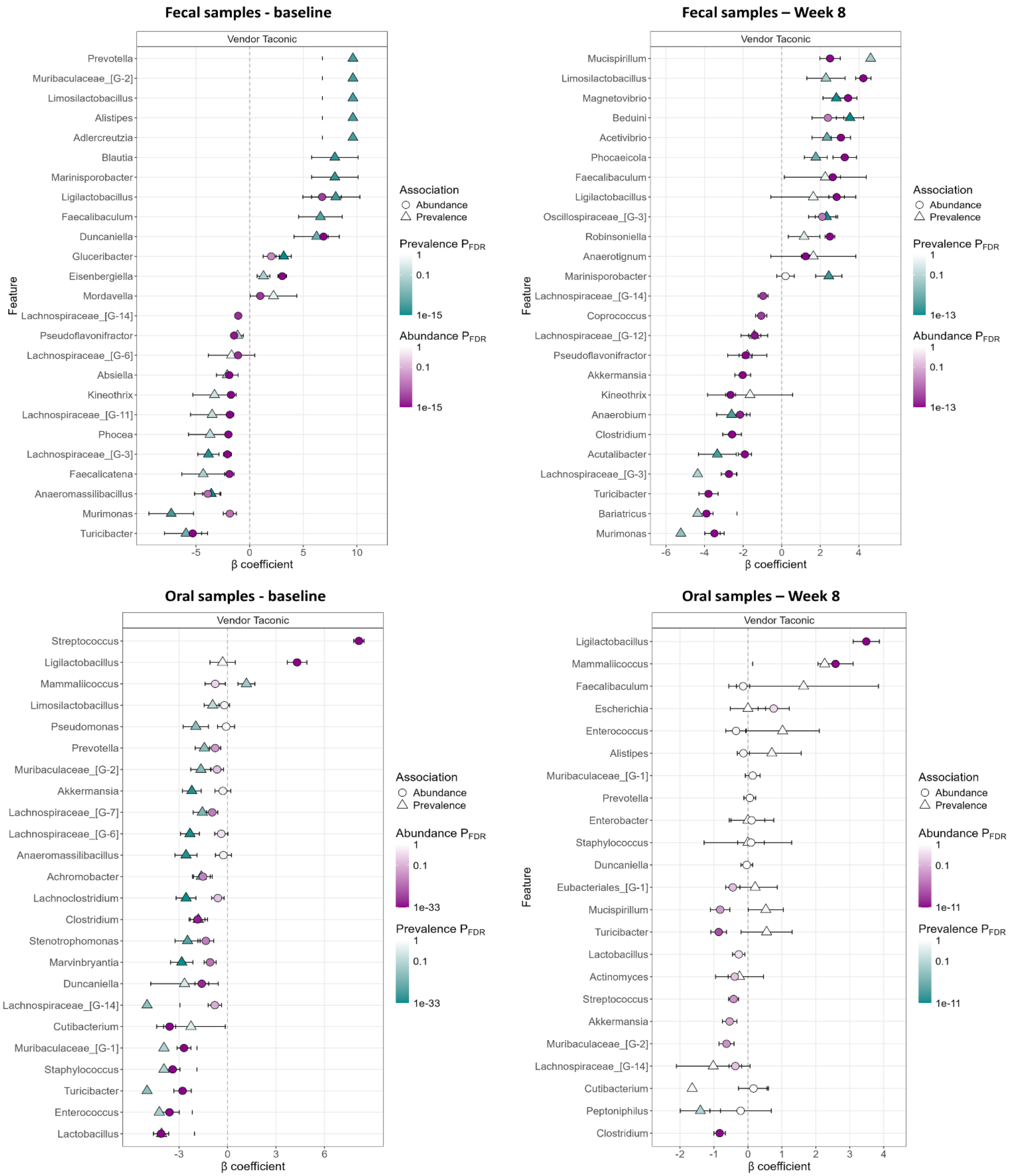

Supplementary Figure 4. Differentially abundant/prevalent genera by vendor (Taconic vs. Jackson) stratified by sample type and 4NQO treatment (time point). Centered log ratio (CLR) transformed data were analyzed with Multivariate Association with Linear Models (MaAsLin3 package in R) to identify differentially abundant genera (FDR ≤ 0.1).

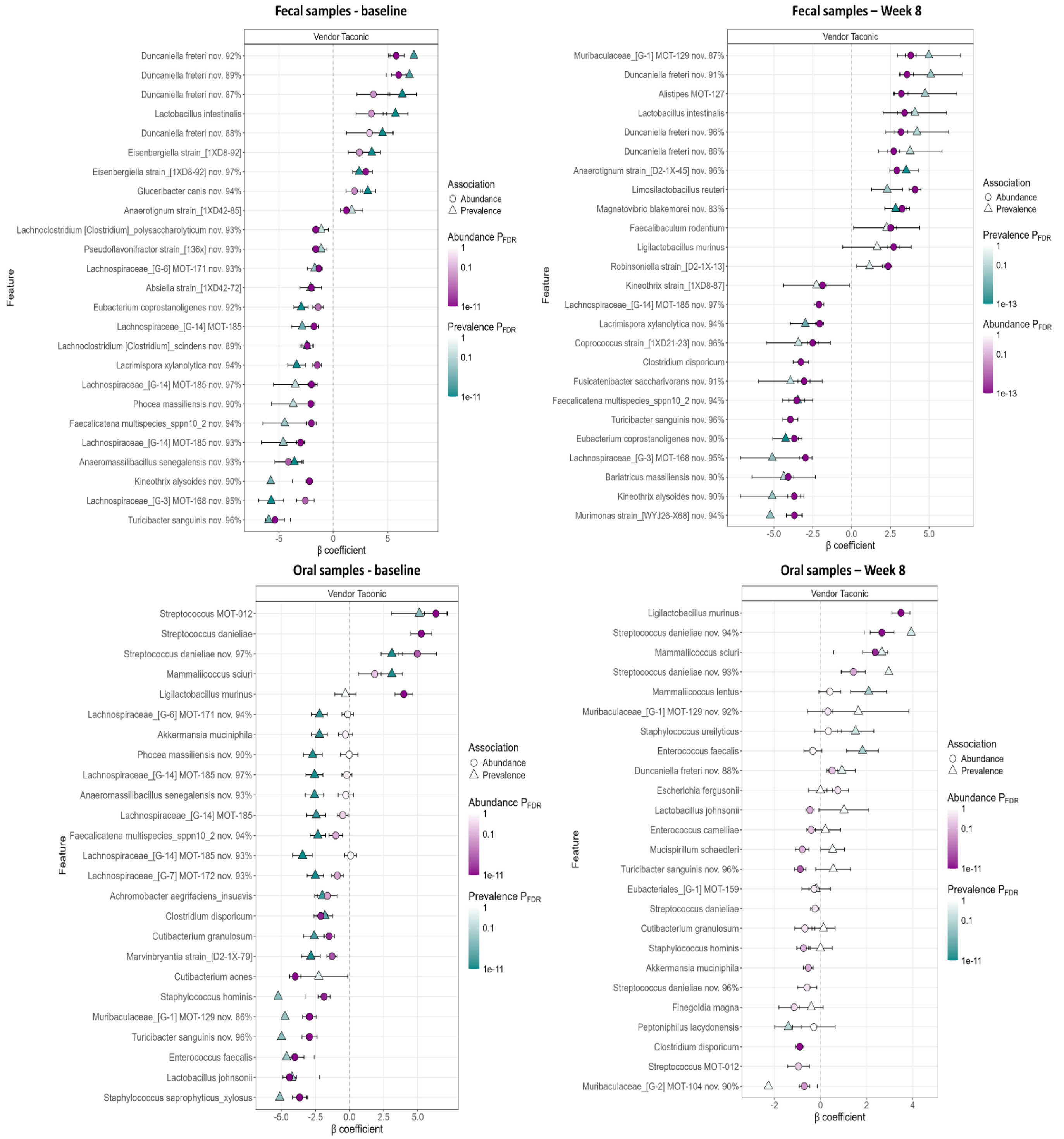

Supplementary Figure 5. Differentially abundant/prevalent species by vendor (Taconic vs. Jackson) stratified by sample type and 4NQO treatment (time point). Centered log ratio (CLR) transformed data were analyzed with Multivariate Association with Linear Models (MaAsLin3 package in R) to identify differentially abundant species (FDR ≤ 0.1).

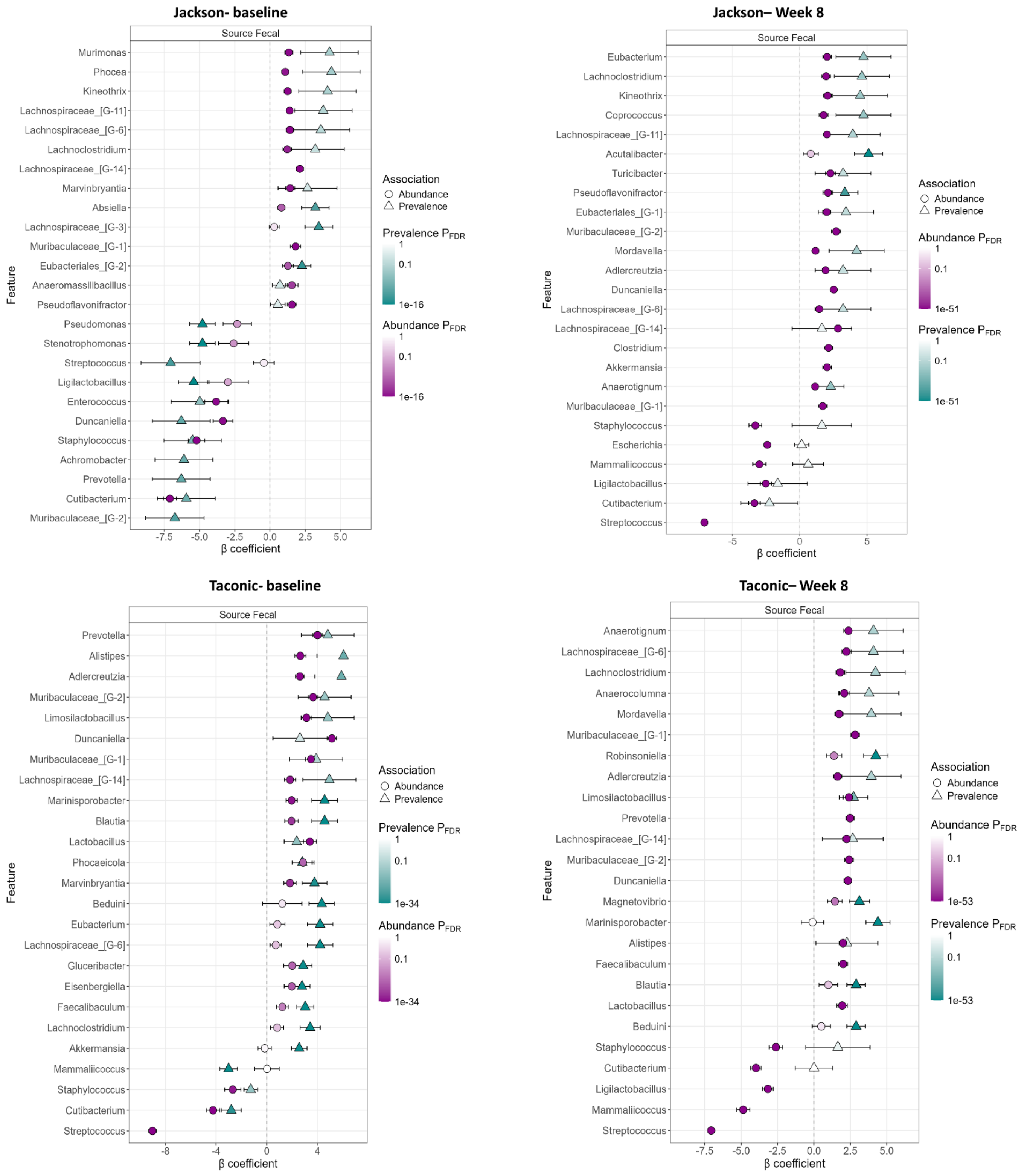

Supplementary Figure 6. Differentially abundant/prevalent genera by sample type (Fecal vs. Oral) stratified by vendor and 4NQO treatment (time point). Centered log ratio (CLR) transformed data were analyzed with Multivariate Association with Linear Models (MaAsLin3 package in R) to identify differentially abundant genera (FDR ≤ 0.1).

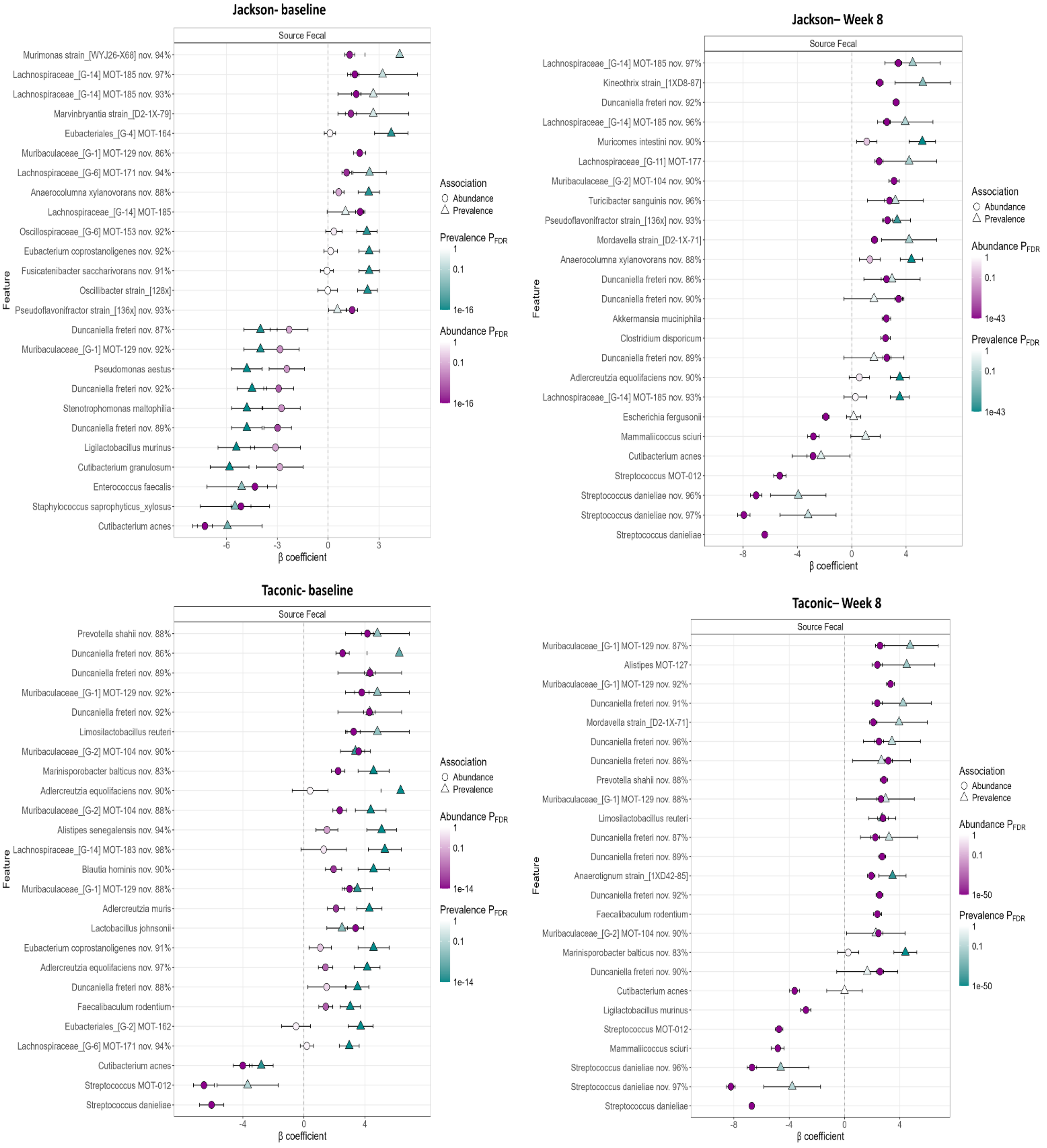

Supplementary Figure 7. Differentially abundant/prevalent species by sample type (Fecal vs. Oral) stratified by vendor and 4NQO treatment (time point). Centered log ratio (CLR) transformed data were analyzed with Multivariate Association with Linear Models (MaAsLin3 package in R) to identify differentially abundant species (FDR ≤ 0.1).

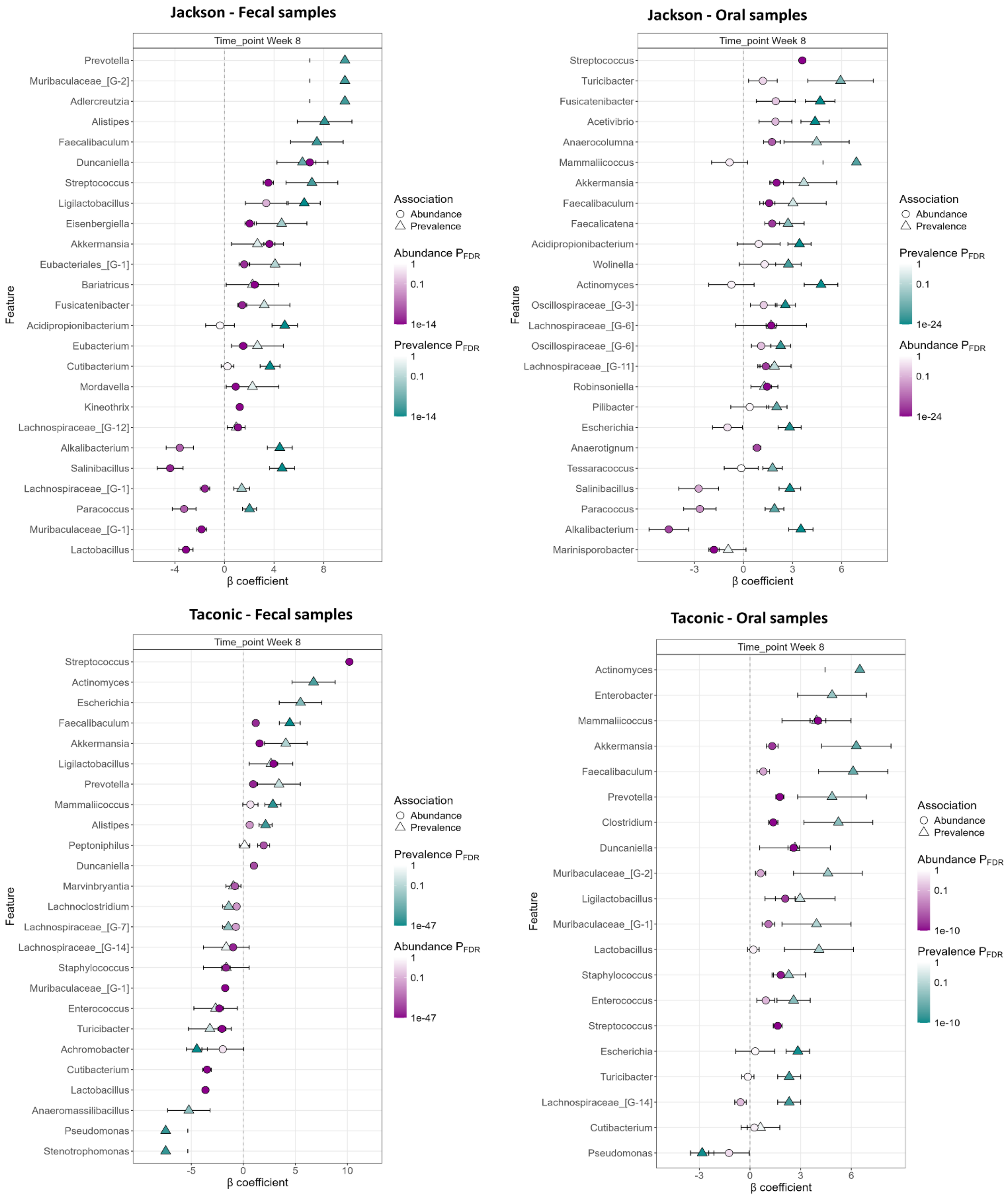

Supplementary Figure 8. Differentially abundant/prevalent genera by 4NQO treatment (Baseline vs. Week 8) stratified by vendor and sample type. Centered log ratio (CLR) transformed data were analyzed with Multivariate Association with Linear Models (MaAsLin3 package in R) to identify differentially abundant genera (FDR ≤ 0.1).

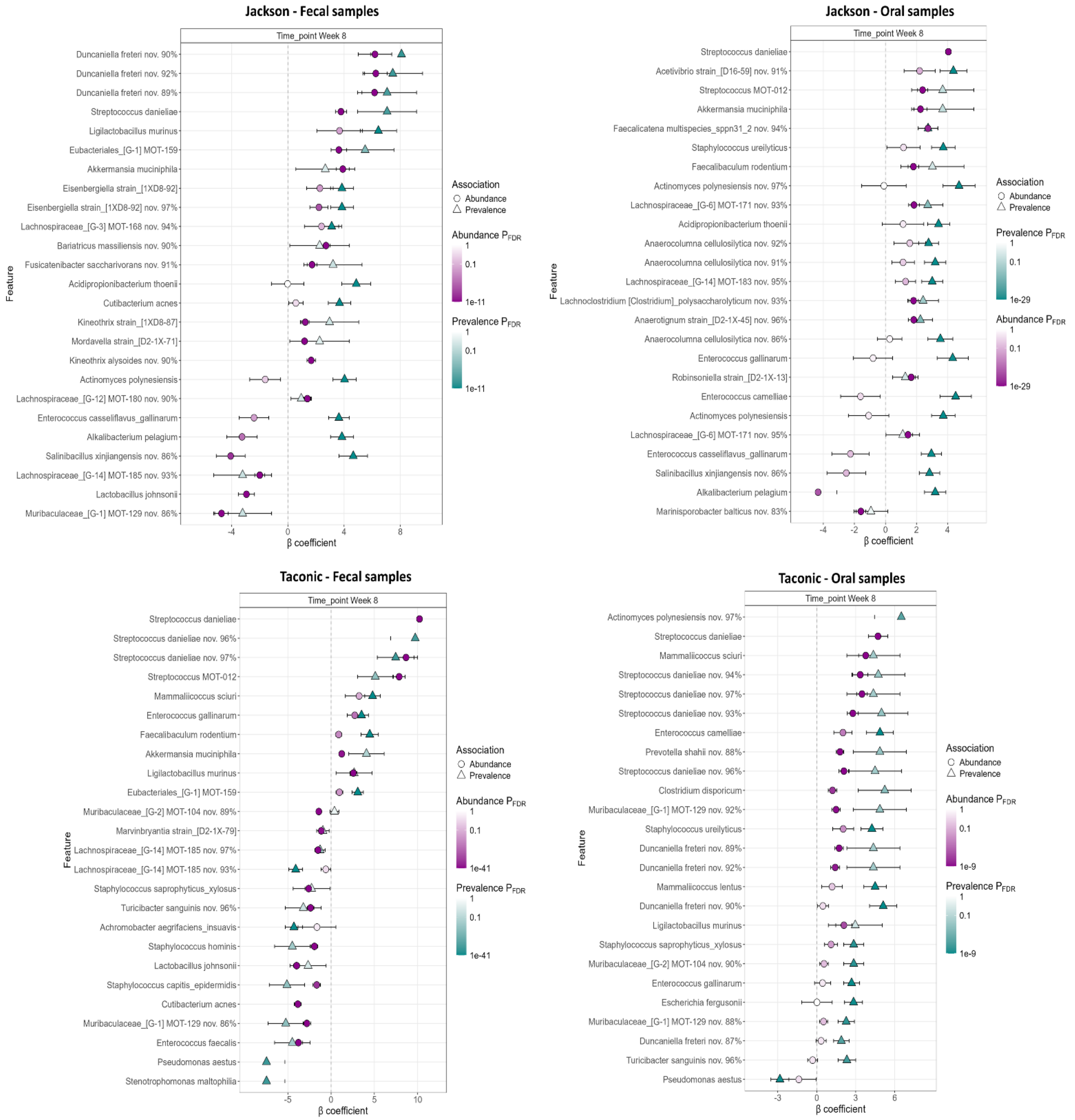

Supplementary Figure 9. Differentially abundant/prevalent species by 4NQO treatment (Baseline vs. Week 8) stratified by vendor and sample type. Centered log ratio (CLR) transformed data were analyzed with Multivariate Association with Linear Models (MaAsLin3 package in R) to identify differentially abundant species (FDR ≤ 0.1).

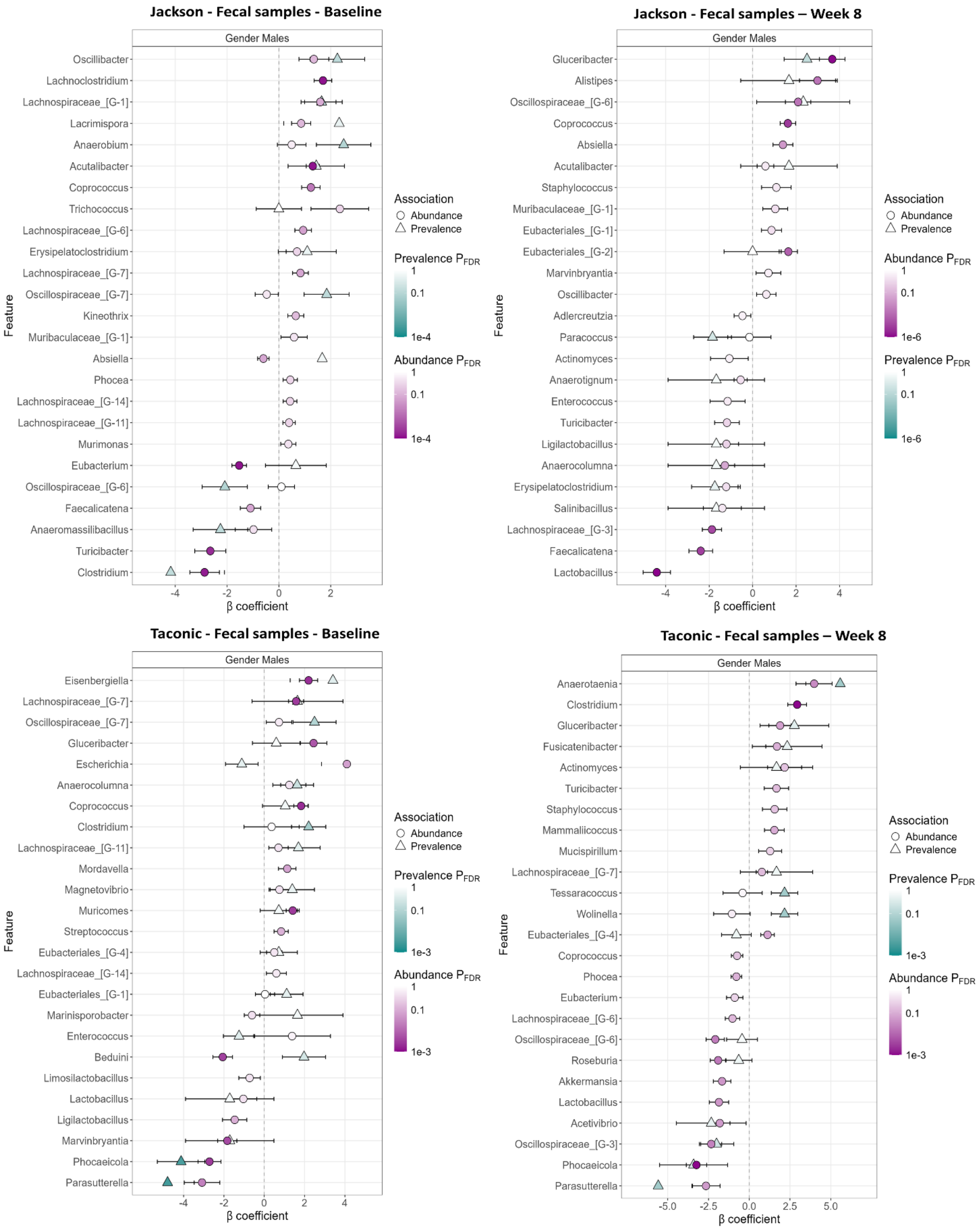

Supplementary Figure 10. Differentially abundant/prevalent genera by sex (Males vs. Females) stratified by vendor and 4NQO treatment (time point) –Fecal samples. Centered log ratio (CLR) transformed data were analyzed with Multivariate Association with Linear Models (MaAsLin3 package in R) to identify differentially abundant genera (FDR ≤ 0.1).

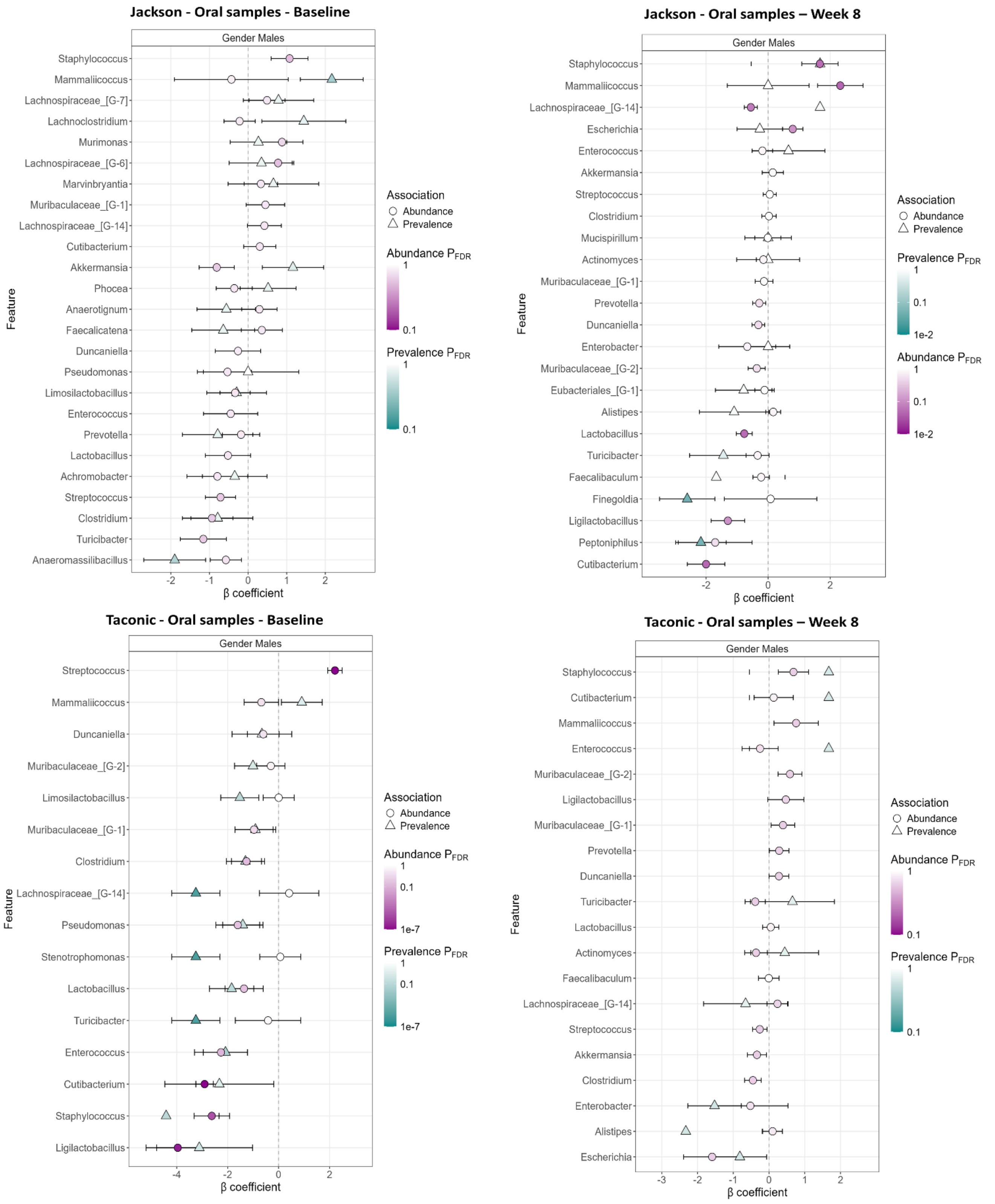

Supplementary Figure 11. Differentially abundant/prevalent genera by sex (Males vs. Females) stratified by vendor and 4NQO treatment (time point) –Oral samples. Centered log ratio (CLR) transformed data were analyzed with Multivariate Association with Linear Models (MaAsLin3 package in R) to identify differentially abundant genera (FDR ≤ 0.1).

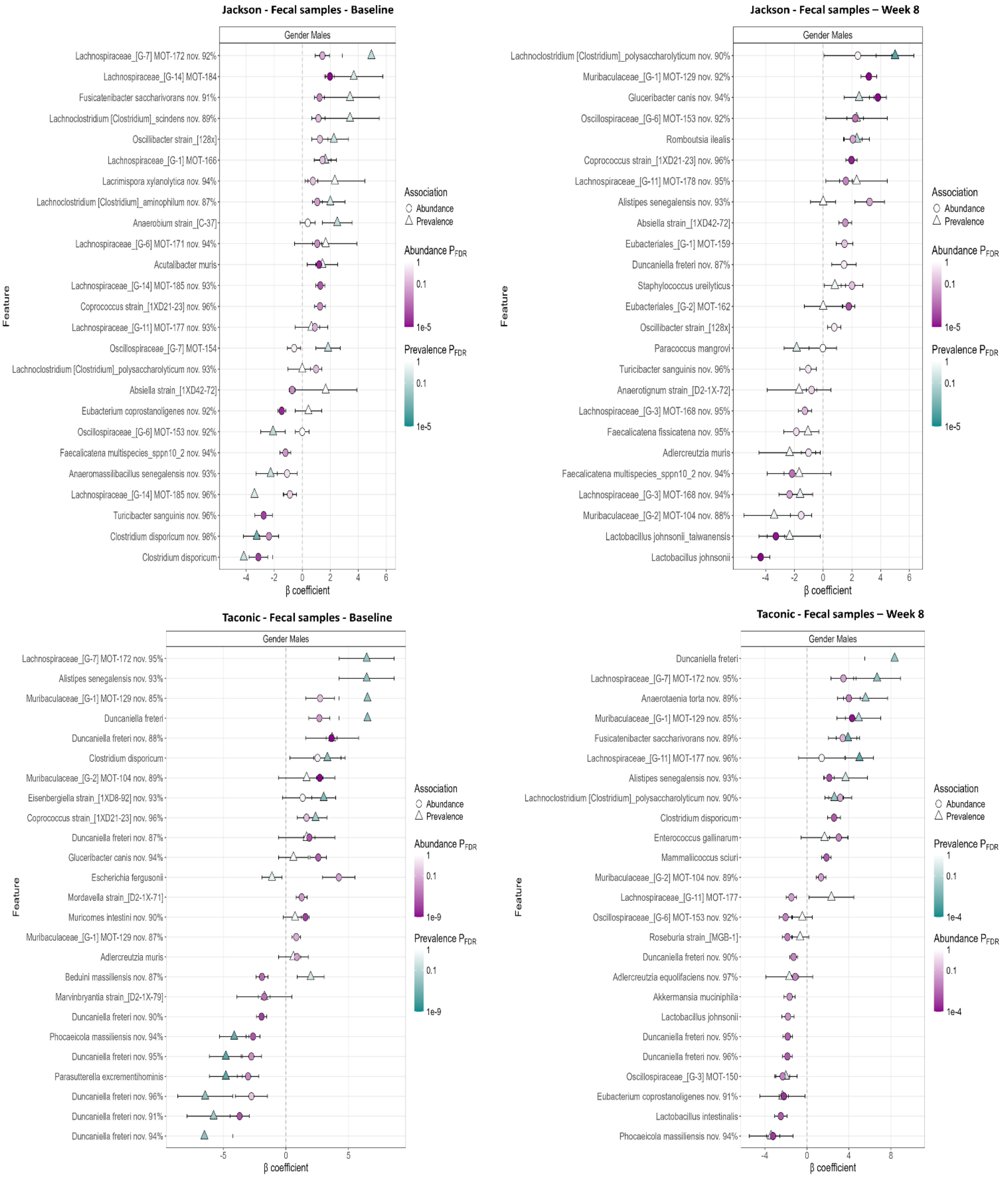

Supplementary Figure 12. Differentially abundant/prevalent species by sex (Males vs. Females) stratified by vendor and 4NQO treatment (time point) –Fecal samples. Centered log ratio (CLR) transformed data were analyzed with Multivariate Association with Linear Models (MaAsLin3 package in R) to identify differentially abundant species (FDR ≤ 0.1).

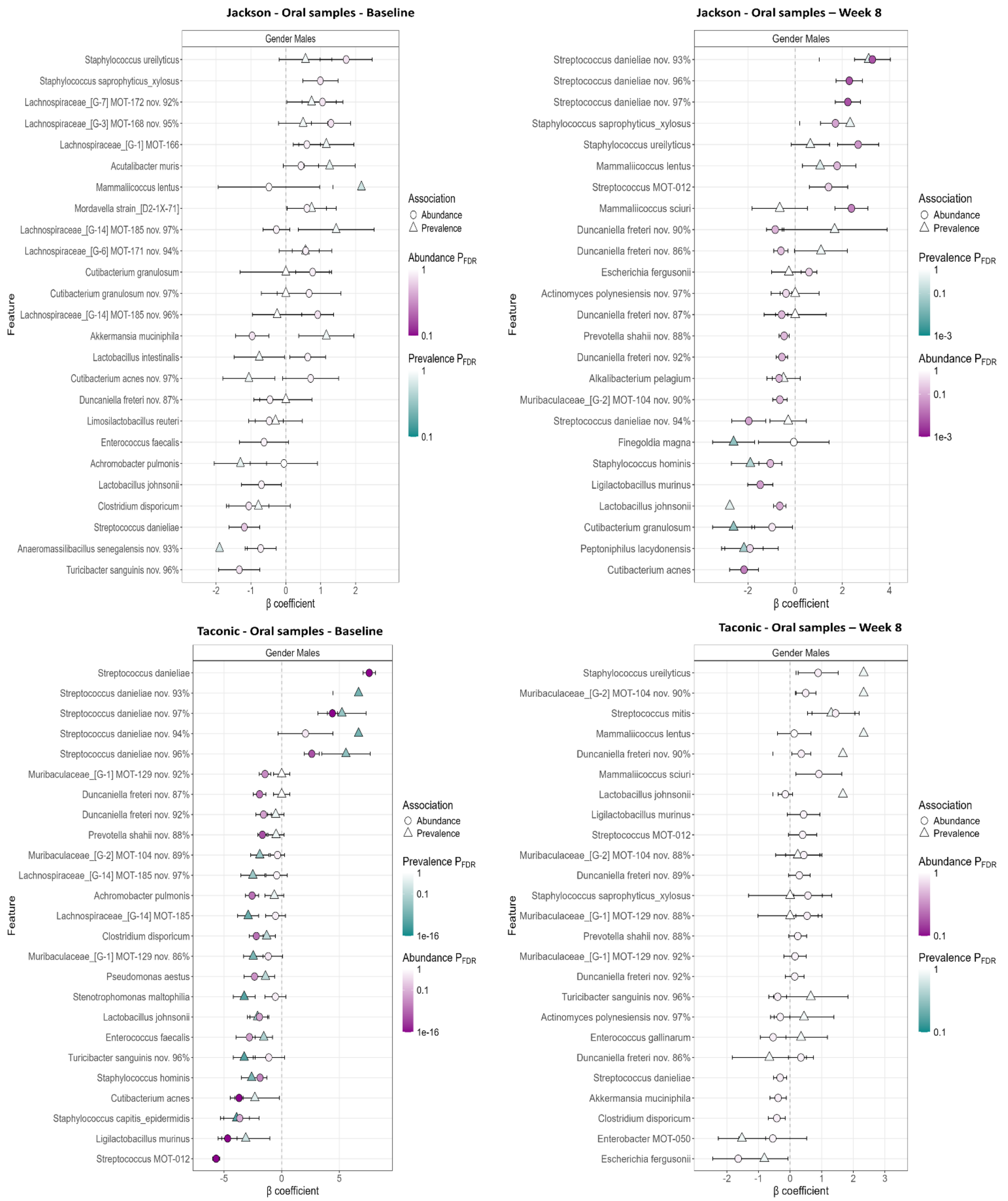

Supplementary Figure 13. Differentially abundant/prevalent species by sex (Males vs. Females) stratified by vendor and 4NQO treatment (time point) –Oral samples. Centered log ratio (CLR) transformed data were analyzed with Multivariate Association with Linear Models (MaAsLin3 package in R) to identify differentially abundant species (FDR ≤ 0.1).
